# *Corydalis saxicola* Bunting total alkaloids attenuate paclitaxel-induced peripheral neuropathy through PKCε/p38 MAPK/TRPV1 signaling pathway

**DOI:** 10.1186/s13020-021-00468-5

**Published:** 2021-07-19

**Authors:** Chu Xue, Si-Xue Liu, Jie Hu, Jin Huang, Hong-Min Liu, Zhi-Xia Qiu, Fang Huang

**Affiliations:** 1 Jiangsu Key Laboratory of TCM Evaluation and Translational Research, Department of Pharmacology of Chinese Materia Medica, China Pharmaceutical University, 639 Longmian Road, Nanjing, 211198 China; 2 Shenzhen Pingle Orthopedic Hospital (Shenzhen Pingshan Traditional Chinese Medicine Hospital), Shenzhen, 518001 China

**Keywords:** Paclitaxel, Paclitaxel-induced peripheral neuropathy, *Corydalis saxicola* Bunting total alkaloids, DRG neurons

## Abstract

**Background:**

*Corydalis saxicola* Bunting, affiliated with the Papaveraceae Juss., has been proven to work well in anti-inflammation, hemostasis, and analgesia. This study was designed to observe the effect and potential mechanism of *Corydalis saxicola* Bunting total alkaloids (CSBTA) on paclitaxel-induced peripheral neuropathy (PIPN).

**Materials and methods:**

Rats were injected 2 mg/kg paclitaxel 4 times and administrated with 30 or 120 mg/kg CSBTA. Mechanical and thermal allodynia and hyperalgesia were tested. After 40 days, serum was collected to detect PGE2, TNF-α, and IL-1β by ELISA. The L4-L6 segment spinal cord, DRG, and plantar skin were harvested, and Western-blot or RT-qPCR analyzed protein and gene levels of pro-inflammatory cytokines, p38 MAPK, PKCε, and TRPV1. The PIPN cell model was established with paclitaxel (300 nM, 5 d) in primary DRG neurons. We examined the effect of CSBTA (25 μg/ml or 50 μg/ml) by measuring the mRNA levels in PGE2, TNF-α and CGRP, and the protein expression on the PKCε/p38 MAPK/TRPV1 signaling pathway in the PIPN cell model.

**Results:**

The results showed that CSBTA effectively ameliorated allodynia and hyperalgesia, and regulated cytokines' contents (PGE2, TNF-α, and IL-1β) and neuropeptides (CGRP and SP) in different tissues in vivo. In addition, CSBTA significantly decreased cytokine gene levels of DRG neurons (PGE2, TNF-α, and CGRP) and the protein expressions of PKCε/p38 MAPK/TRPV1 signaling pathway in vivo and in vitro.

**Conclusion:**

Therefore, CSBTA has a perspective therapeutic effect on the treatment of paclitaxel-induced peripheral neuropathy.

## Introduction

Paclitaxel, extracted from *Taxus brevifolia* Nutt., is widely used to treat solid tumors, such as breast cancer, lung cancer, ovarian cancer [1]. It exerts anticancer effects by increasing the stability of tubulin polymer and inhibiting the proliferation of cancer cells. However, paclitaxel-induced peripheral neuropathy (PIPN), serious side effects during paclitaxel treatment, severely limits its application in the clinic and is characterized by numbness, tingling, spontaneous pain, and temperature sensitivity. The incidence of PIPN significantly increases during long-term treatment, and this side effect will persist for months or years [2]. PIPN responds poorly to clinical analgesics such as opioid analgesics, anticonvulsants, or antidepressants [3]. There are no proper prevention and treatment measures, and the underlying mechanism is still unclear.

The molecular mechanisms of pathogenesis have been investigated recently. It is widely recognized that mitochondrial dysfunction is a significant contributor to PIPN. In addition, paclitaxel could promote cell damage by inhibiting tubulin depolymerization, interfering with the stability of microtubules (including neuronal axons), switching on mitochondrial permeability transition pore (mPTP), disturbing calcium signals in mitochondria and endoplasmic reticulum, and so on. The chemotherapeutic drug-induced inflammatory process is also considered a potential factor. The release of pro-inflammatory cytokines and chemokines is regarded as one of the main mechanisms of neuro-immune regulation [4, 5].

Currently, some studies have emphasized the role of transient receptor potential vanilloid type 1 (TRPV1) in the pathological progress of PIPN [6–8]. TRPV1, one of the nonselective cation channels, could be activated by heat (43 ℃), protons, and ligands (e.g., capsaicin) [6]. More and more studies have shown that TRPV1 is involved in the essential mechanisms associated with inflammatory and neuropathic pain [9–11]. Hara et al*.* have confirmed that intraperitoneal injection of paclitaxel could increase the TRPV1 level in rat plantar skin and sensitize the TRPV1 channel, which is regarded as the cause of heat hyperalgesia [7]. Some studies have shown that intraperitoneal injection of TRPV1 antagonists (such as capsazepine and SB366791) in PIPN rats would attenuate paclitaxel-induced thermal hyperalgesia in a dose-dependent manner but did not exhibit a pronounced effect on mechanical allodynia [6], further confirming the relationship between TRPV1 and PIPN thermal hyperalgesia. The activation of TRPV1 leads to depolarization of neurons and causes the production and release of neuropeptides such as CGRP and SP in peripheral and central nerve endings. Neuropeptides activate their corresponding receptors and enhance the sensitivity of nociceptors [12].

Many signaling pathways are involved in the pathological process of TRPV1 activation in the PIPN process. PKC and mitogen-activated protein kinase (MAPK) signaling cascades are considered essential in regulating TRPV1. TRP channels are often considered to be downstream of PKC signaling and linked with paclitaxel-induced pain behavior. Paclitaxel has a lipopolysaccharide (LPS)-like effect, which increases intracellular Ca^2+^ levels in DRG and promotes phosphorylation of PKC. Increased PKC activity in DRG was observed in PINP mouse models, and inhibition of PKC attenuated paclitaxel-induced peripheral neuropathy [13]. Studies have shown that paclitaxel could activate proteinase-activated receptors 2 (PAR2) through the PLC-PKC pathway and lead to TRPV1, TRPV4, and TRPA1 sensitivity, and eventually produce allodynia and hyperalgesia. In a variety of PKC subtypes, the importance of PKCε activation has been proved in paclitaxel-induced mechanical pain. However, PKCε v1-2 (PKCε inhibitor) has only a modest effect on reducing hyperalgesia [14].

MAPK is widely reported as the downstream of the PKC signal and its regulatory effect on TRPV1 has been studied [15]. MAPK family is a class of highly conserved serine/threonine protein kinases, while MAPK is mediated by p38 rather than ERK in the pathogenesis of pain [16]. p38 MAPK, as a tyrosine phosphoprotein kinase, is activated by stress signals and inflammatory stimuli and contributes to cellular responses associated with neuropathic pain [17, 18]. Activation of p38 MAPK in DRG can increase translation and transport of TRPV1 to the terminal of the peripheral nociceptor, contributing to the maintenance of thermal hyperalgesia [16]. In general, p38 MAPK plays a crucial role in dorsal root ganglion neurons and spinal cord neurons and participates in the pathogenesis of PIPN [19]. The expression of ERK1/2 and p38 MAPK was increased in DRG tissues, and administration of SB203580 (p38 MAPK inhibitor) prevented but could not reverse paclitaxel-induced pain behavior in PIPN rat models [20]. The effect of p38 MAPK activation was limited to hyperalgesia and had no effect on mechanical hypersensitivity, which is consistent with the critical role of TRPV1 as a p38 MAPK downstream target [21].

*Corydalis saxicola* Bunting, affiliated with the Papaveraceae Juss., is the natural grass of the perennial herb. Given the function of clearing away heat, detoxifying dampness, relieving pain, and stopping bleeding, *Corydalis saxicola* Bunting has been widely applied in hepatic diseases such as hepatitis, liver cirrhosis, and liver cancer in the clinic [22]. *Corydalis saxicola* Bunting consists of a variety of protoberberine type I alkaloids, including dehydrocavidine, berberine, tetrahydropalmatine, (±) cavidine, (±) tetrahydropalmatine, and protopine, which are called *Corydalis saxicola* Bunting total alkaloids (CSBTA) [23, 24]. Subcutaneous injection of 50 mg/kg CSBTA tended to restore the writhing response in mice and had a significant inhibitory effect when the dose reaches 100 mg/kg. 100 mg/kg CSBTA also increased the threshold for thermal stimulation in the tail-flick test [25]. Our group has previously found that oral administration of CSBTA (30 mg/kg, 60 mg/kg, and 120 mg/kg) relieved cisplatin-induced neuropathic pain by decreasing the phosphorylation p38 MAPK and downstream TRPV1 [25]. CSBTA also suppressed RANKL-induced NF-κB and c-Fos/NFATc1 pathways to attenuate Walker 256-induced bone pain [26]. However, there is no report on the therapeutic function or molecular targets of CSBTA in PIPN. Based on current studies, it is assumed that the PKCε/p38 MAPK/TRPV1 signaling pathway may participate in the pathological process. Therefore, the purpose of this study was to observe the therapeutic effect of CSBTA on PIPN and explore its mechanism by animal and cell experiments.

## Materials and methods

### Reagents

The commercial paclitaxel injections (Paclitaxel Injection®, Lot: H99404910) were purchased from SichuanTai Ji Group Co., Ltd (Chengdu, China). CSBTA (Yanhuanglian total alkaloids capsules, Lot: 160701) was kindly supplied by Nanjing Zhongshan Pharm Co., Ltd (Nanjing, China). Sodium carboxymethyl cellulose (CMC-Na) was purchased from Sinopharm Chemical Reagent Co., Ltd (Shanghai, China). CSBTA powder was suspended in CMC-Na solution in advance. The quality specification of the CSBTA capsule is controlled by the content of total alkaloids (calculated by dehydrocavidine) and the amount of dehydrocavidine, palmatine, and berberine. Referring to the standard sample of dehydrocavidine, palmatine, and berberine (supplied by National Institutes for Food and Drug Control, Beijing, China), the total alkaloids fraction was analyzed as 58% by HPLC–UV analysis.

### Animals

Adult male Sprague–Dawley rats (180 ± 20 g) were supplied by the Laboratory Animal Center of Nanjing Qinglongshan (Agreement Number: SCXK-zhe-2014-0001). All rats were maintained under the standard conditions in China Pharmaceutical University Laboratory Animal Center under the12 h dark–light cycles in the humanized environment (temperature, 23 ± 2 °C, and humidity, 50 ± 10%) with regular chow diet and tap water ad libitum. After acclimation for a week, the rats were used for the following experiments.

### Grouping and administration

Rats were randomly divided into 4 groups: blank group (physiological saline & 0.5% CMC-Na solution), paclitaxel group (2 mg/kg paclitaxel & 0.5% CMC-Na solution), 30 mg/kg CSBTA group (2 mg/kg paclitaxel & 30 mg/kg CSBTA suspended in CMC-Na solution) and 120 mg/kg CSBTA group (2 mg/kg paclitaxel & 120 mg/kg CSBTA suspended in CMC-Na solution). Except for the blank group, rats were intraperitoneally injected with 2 mg/kg paclitaxel (Paclitaxel Injection®, Tai Ji, Chengdu, China) at 1st, 3rd, 5th, 7th days, and the blank group was intraperitoneally given the corresponding volume of physiological saline [27–29]. From the first day of the experiment, oral administration of CSBTA with different concentrations (30, 120 mg/kg) was given [25]. At the same time, the blank group and the model group were administered with a corresponding volume of 0.5% CMC-Na solution. During the experimental period, the body weights were recorded every day before drug administration to evaluate the potential effect of CSBTA on body weight changes.

Ahead of the final experiments, the food was deprived overnight (free access to water). After mice were anesthetized, blood was collected from the abdominal aorta, centrifuged at 3000 rpm for 10 min to be harvested plasma to determine PGE2, TNF-α, and IL-1β. L4-L6 segment spinal cord, DRG, and plantar skin were harvested and immediately stored at − 80 ℃ till analysis.

### Mechanical hyperalgesia—Von-Frey test

The mechanical pain threshold of rats was detected by Von-Frey filament according to the "up and down" method initiated by Chaplan [30]. As described previously [25], the rats were individually placed in a transparent plexiglass box with a cover at the top and a reticular structure at the bottom. Every rat was subjected to an adaptive test 1 week before the formal experiment. After adaptation for 15 min, the mechanical pain threshold was measured when rats were in a peaceful state, and the movement of walking and scratching disappeared. Von-Frey filament (Stoelting Company, Chicago, USA, stimulus intensity range: 4–180 g) was used to prick the middle area of the ipsilateral posterior toe through the bottom mesh. The fiber needle vertically pressed the plantar skin until it was warped into a "C" shape. If the hind paw's retraction, movement, or lameness appeared, a positive reaction "X" was designated and recorded the corresponding value. And then, the strength nearest to low-intensity was used to stimulate, and negative reaction "O" was recorded if there were no response after stimulus for more than 5 s. Sequentially, a closest higher intensity was continued to stimulate till the rats showed positive behavior. Stimulation was conducted every 2 min and repeated six times. A double-blind trial tested mechanical hyperalgesia. Followingly, we could obtain the "X" and "O" sequences and finally calculated the mechanical pain threshold. The mechanical pain threshold test in each rat was repeated three times to calculate the average response.

### Thermal sensitivity—laser thermal pain meter

PL-200 laser thermal pain meter (PL-200, Taimeng Technology, Chengdu, China) was used to detect heat hyperalgesia to assay the latent period of heat-stimulated retraction reflex. As described previously [25], each animal was subjected to an adaptive test 1 week before the formal experiment. Under constant ambient temperature, rats were placed in an observation box for 15 min in advance to make them accommodative and tranquil. Afterward, the optical source (intensity at 35%) was moved to plantar, and the time (from the initial irradiation) was recorded when thermal pain reaction (contracting claw, licking foot, and scratching) occurred. The cutoff time was 25 s to prevent possible injury from high temperature. The thermal sensitivity test was repeated three times at least 5 min intervals in each rat, and the average was applied for data analysis. A double-blind trial tested thermal sensitivity.

### Thermal sensitivity—tail immersion assay

Half an hour after drug administration, rats were subjected to a tail-flick test. As described previously [27], each animal was subjected to an adaptive test 1 week before the formal experiment. After the rat was no longer struggling, the tail was immersed into a water bath, and we recorded the time from immersion to tail flick reaction. The temperature was set at 47 ℃, adopted by the tail-flick time lasting 10 s for most rats. To prevent the injury from high temperature, we selected 20 s as a cutoff time. Each rat was tested three times with at least 5 min intervals, and the average of the three tests was used for data analysis. A double-blind trial tested thermal sensitivity.

### Thermal sensitivity—cold hyperalgesia

Similarly, rats were subjected to a cold hyperalgesia test 30 min after drug administration. As described previously [31, 32], we used dry ice to assay the cold pain threshold of rats, and the results were expressed as the first occurrence time of paw withdrawal, lameness, scratching, or other reactions in rats after cold stimulation. The room's temperature was kept stable, and rats were placed in an observation box for 15 min to make them quiet. After the dry ice placing on plantar, we recorded time from the beginning to the emergence of contracting claw, licking foot, or scratching. The cutoff time was 90 s to avoid skin cold injury. Each animal was subjected to an adaptive test 1 week before the formal experiment. A double-blind trial tested cold hyperalgesia.

### TNF-α, IL-1β, and PGE2 in serum and plantar skin tissues

After 12 h of the 40th day, rats in each group were fasted for 10 h (free access to water) and anesthetized. Blood was rapidly withdrawn from the abdominal aorta to clean EP tubes. Serum was harvested after centrifugation at 3000 rpm for 10 min. The harvested plantar skin tissues from corresponding rats were immediately shaved on the ice. A piece of 1 g tissue was collected and cut into pieces. Subsequently, they were soaked in 5 ml physiological saline after a gentle vortex at 4 ℃ for 2 h. Afterward, the mixtures were centrifuged at 3000 rpm for 10 min at 4 ℃, and the harvested supernatant was stored at − 80 ℃ till analysis [25]. The levels of TNF-α, IL-1β, and PGE2 in obtained plantar skin supernatant and serum were separately detected by ELISA (Calvin Biotechnology, Suzhou, China) according to kit instructions.

### Preparation and quality control of *Corydalis saxicola* Bunting total alkaloids

**Fig. 1:**
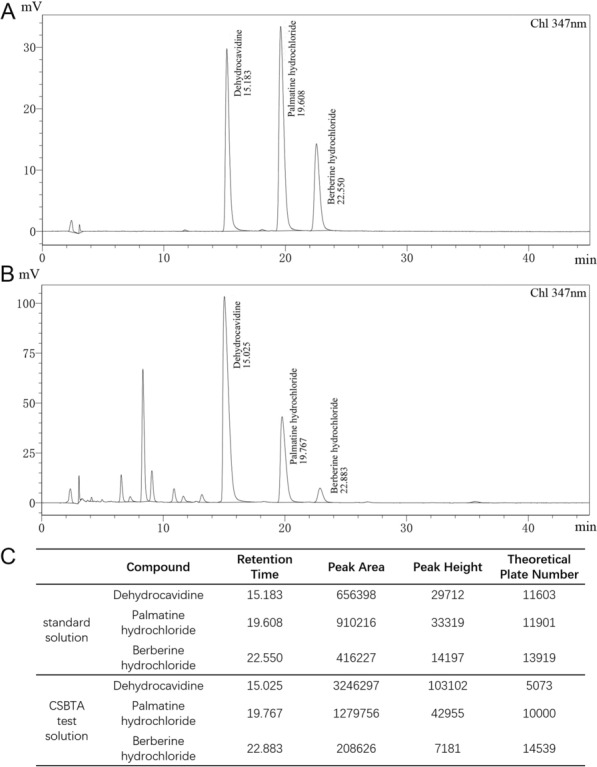
HPLC chromatograms of CSBTA. **A** HPLC chromatograms of the standard solution was detected at 347 nm. The retention time of dehydrocarvidine, palmatine hydrochloride, and berberine hydrochloride were 15 min, 19.6 min, and 22.5 min, respectively. **B** HPLC chromatograms of CSBTA test solution detected at 347 nm. **C** HPLC chromatograms information of standard solution and CSBTA test solution detected at 347 nm. The contents of dehydrocarvidine, palmatine hydrochloride, and berberine hydrochloride in CSBTA were 19.8%, 5.6%, and 1%, respectively

*Corydalis saxicola* Bunting total alkaloids (CSBTA) were provided by Nanjing Zhongshan Pharmaceutical Co., Ltd. (Batch number: 191201). According to China Pharmacopoeia (2005 version), the composition of CSBTA was determined by high-performance liquid chromatography (Appendix VI D) [26, 33]. In brief, the quantitative analysis of CSBTA by high-performance-liquid chromatography (HPLC) was performed by SHIMADZU LC-20 AT HPLC system (SHIMADZU, Japan) equipped with a quaternary ammonium salt pump solvent management system, an online degasser, and an autosampler. The separation was conducted by an Agilent ZARBAX SB-C18 column (the USA, 4.6 × 250 mm, 5 μm) with the 10 μl injection volume, and the column temperature was maintained at 30 °C. The mobile phase was composed of acetonitrile (A) and potassium dihydrogen phosphate solution (B, 0.01 mol/l), and gradient elution was set as 25% A: 75% B with the 1 ml/min flow rate. The detection was performed as 347 nm. A mixed solution of dehydrocarvidine 20 μg, palmatine hydrochloride 20 μg, and berberine hydrochloride 10 μg was prepared in 1 ml methanol as a standard solution. The CSBTA test solution consists of 25 mg CSBTA dissolved in 50 ml methanol. The representative chromatography was depicted in Fig. 1. For the in vitro experiments, 50 mg CSBTA powder was dissolved in 1 ml DMSO as stock solution.

### Primary DRG neurons cell culture

As described previously [34, 35], the back skin of rats was quickly cut with surgical scissors to isolate the spine after anesthesia. DRG tissue was extracted and placed in a MEM medium tube (Gibco, USA) without fetal bovine serum (FBS, Gibco, USA). Subsequently, an aliquot of 2 ml collagenase A (Roche, USA, 1 mg/ml) was added to digest cells for about 90 min. Then, the digestion solution was replaced by 0.25% trypsin (Gibco, USA) for another 20 min. Finally, the digestion was terminated by the MEM medium containing 10% FBS. MEM medium was mechanically triturated with a 1 ml pipette until the solution turned milky. After filtering through a 70 μm cell strainer, the solution was centrifuged at 1000 rpm for 5 min. The dissociated neurons suspended in MEM medium containing 10% FBS and 1% penicillin and streptomycin mixture (Gibco) were plated onto poly-L-lysine-coated 6-well plates (Corning Life Sciences, Acton, MA) at a density of 4 × 10^5^ cells/well and incubated at 37 °C in a 5% CO2 and 95% humidity atmosphere. After culturing for 24 h, MEM medium was replaced with neuronal growth medium (Neurobasal medium: B27 = 50:1, both purchased from Gibco, USA), and 1% other agents including penicillin and streptomycin mixture (Gibco), L-Glutamine (0.1 mg/ml, Sigma-Aldrich), and cytarabine (5 μg/ml, Sigma Aldrich) was added. Medium with supplements was changed every two days. The cells were used for experiments on the 4th-10th days.

### Cell viability assay

CSBTA group was treated with 0, 0.05, 0.5, 5, 50 μg/ml of CSBTA, and paclitaxel groups were given 0, 1, 10, 100, 500, 1000 nM paclitaxel (A4393, APExBIO, USA) on the 4th day of primary DRG neurons. Five days after administration, a Cell Counting Kit-8 (CCK-8) solution (Dojindo, Japan) was added. After incubation for 4 h, absorbance at 450 nm was measured with a microplate reader.

### Drug treatment

Primary DRG neuron cells were divided into the following five groups for the experiment on the 4th day: blank group (phosphate buffer saline), paclitaxel group (300 nM paclitaxel), 25 μg/ml CSBTA group (300 nM paclitaxel + 25 μg/ml CSBTA), 50 μg/ml CSBTA group (300 nM + 50 μg/ml CSBTA) and PKCε inhibitor group (300 nM paclitaxel + 100 nM Staurosporine (A8192, APExBIO, USA)). Staurosporine group was regarded as positive control. In some trials, 1 μM SB203580 (HY-10256, MCE) was administered as an inhibitor of p38 MAPK. Five days after administration, cells were collected for subsequent experiments.

### Western blot analysis

As described previously [25], the spinal cord and DRG were lysed in lysis buffer (RIPA: PMSF: phosphatase inhibitor = 100:1:1) at 1:5 (mg·μ1^−1^) and vigorously crushed by glass homogenizer on ice. An appropriate aliquot of lysis buffer was added, and cell scrapers on ice cells scrapped cells. The homogenates were immediately centrifuged at 12,000 rpm for 20 min at 4 ℃. Protein quantification was performed according to the Bicinchoninic Acid Protein Assay kit (Nanjing Jiancheng Bioengineering Institute, Nanjing, China). Subsequently, extracted protein samples (6 × loading buffer: supernatant = 1:2, v/v) were boiled for 15 min and stored at − 80 ℃ till analysis.

Loading protein (50 μg) was electrophoresed and separated on a 10% SDS–polyacrylamide gel (SDS-PAGE) and transferred to a polyvinylidene difluoride (PVDF) membrane (0.22 μm, Pall, USA). PVDF membrane was blocked in 5% skimmed milk in TBST buffer (Tris–HCl, 5 mM, pH 7.6, NaCl 136 mM, 0.05% Tween-20) at room temperature for 2 h, followed by incubation with different primary antibodies—β-actin (1:5000, Bioworld, USA), p38 (1:1000, Abcam, USA), p-p38 (1:1000, Cell Signaling Technology, USA), PKCε (1:1000, Abcam, USA) and TRPV1 (1:200, Alomone, Israel) in TBST at 4 ℃ overnight. After being washed with TBST three times, membranes were further incubated with horseradish peroxides (HRP)-conjugated secondary antibody (1:5000 dilution in TBST) for 2 h at room temperature. Antibody binding was detected by enhanced chemiluminescence (ECL). The β-actin was used as an endogenous reference. Imagines were acquired by Bio-Rad and quantified by densitometry analysis using Image J software.

### Real-time qPCR analysis

Spinal cord and DRG tissue weighing 100 mg were homogenized in 1 ml Trizol reagent (Invitrogen, USA) on ice to extract total RNA. Cells were lysed with 1 ml Trizol reagent per well to extract total RNA [36]. RT-PCR was proceeded according to HieffTM First Strand cDNA Synthesis SuperMix Kit instruction. The β-actin was used as an endogenous reference. Quantitative PCR was performed in 20 μl reactions using HieffTM qPCR SYBR Green Mester Mix (No Rox) kit. Primer sequences are listed in Table 1. The ratios between the candidate genes and β-actin mRNA were detected by BioRad image system (Bio-Rad, USA) and calculated by the comparative threshold cycle (Ct) method.

**Table 1:** The primers for the quantitative RT-PCR

Gene	Forward primer	Reverse primer
TRPV1	AGAAGGGGAACCAGGGCAAAG	TCAACGAGGACCCAGGCAACT
PKC epsilon	TCTGGAAGCAGCAATAGAGTT	TCATCAAGGTGTTAGGCAAAG
Tac1	CTCACAAAAGGCATAAAACAGATT	TGAATAGATAGTGCGTTACAGGGTT
CGRP	GTCCCTCCTCTCCTTTCCAGTT	AGATTCCAGATACCATCCTTTGCC
PGE2	AGGGAGCATACAGCGAAGGTG	TGCGGATTGTCTGGCAGTAGC
TNF-α	GCGTGTTCATCCGTTCTCTACC	TACTTCAGCGTCTCGTGTGTTTCT
β-actin	ATCATTGCTCCTCCTGAGCG	CGCAGCTCAGTAACAGTCCG

### Immunofluorescence staining

After paclitaxel and CSBTA treatment, primary DRG neurons were fixed with 4% paraformaldehyde and then blocked with 5% BSA for 1.5 h. After incubation with primary antibody (anti-PKCε, 1:100) overnight at 4 °C, cells were incubated with fluorescein isothiocyanate-conjugated secondary antibody for 2 h at 37 °C. Nuclei were stained with DAPI (Beyotime Institute of Biotechnology, Shanghai, China). The immunofluorescence images were visualized under a confocal scanning microscope (Zeiss LSM 700, Jena, Germany).

### Statistical analysis

Data were expressed as a mean ± SD (standard deviation) of at least three independent experiments. Statistical analysis was performed using GraphPad Prism software, version 6.0 (San Diego, CA, USA). The statistical significance was analyzed using unpaired Student’s t-test, or one-way ANOVA followed by Dunnett’s multiple comparison test. A value of p < 0.05 was considered to be statistically significant.

## Results

### The purity of dehydrocarvidine, palmatine hydrochloride, and berberine hydrochloride in CSBTA

As shown in Fig. 1, the retention time of dehydrocarvidine, palmatine hydrochloride, and berberine hydrochloride were about 15 min, 19.6 min, and 22.5 min, respectively. The CSBTA sample had no abnormal peak at the appropriate location. The results showed that the contents of dehydrocarvidine, palmatine hydrochloride, and berberine hydrochloride in CSBTA were 19.8%, 5.6%, and 1%, respectively.

### CSBTA normalized the neurological behaviors in PIPN rats

**Fig. 2:**
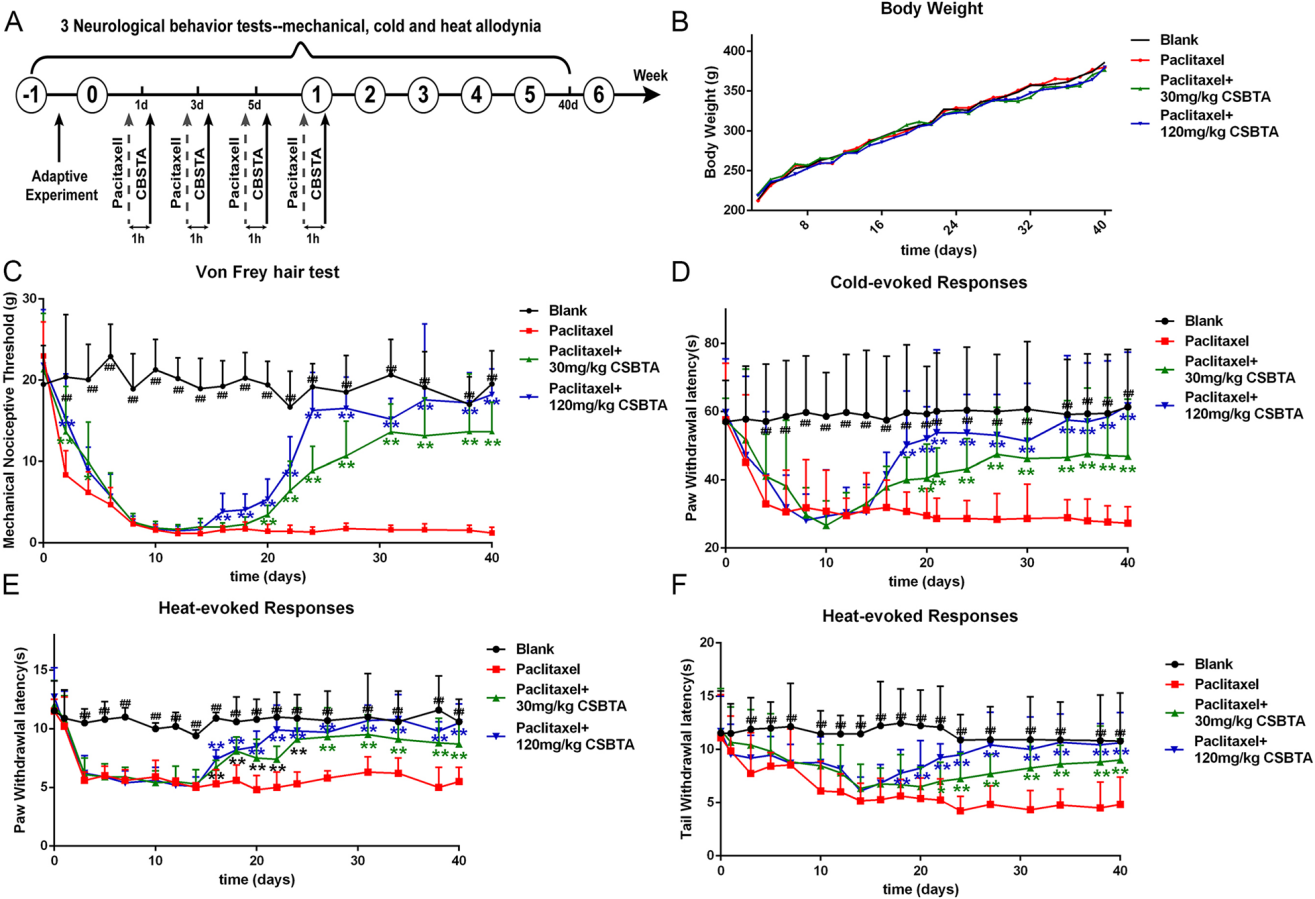
The neuroprotective effects of CSBTA in the PIPN model. **A** In the first week, 2 mg/kg paclitaxel was injected intraperitoneally every other day. Vehicle or CSBTA (30 mg/kg or 120 mg/kg) were oral administrator before paclitaxel treatment. **B** Mean body weight during the experimental period. **C** The Von Frey filament test was used to detect the change of mechanical allodynia in rats. The lower the value, the higher the sensitivity of the animal to mechanical stimulation. **D** Drikold simulation experiment was used to detect cold hyperalgesia. The lower the value, the higher the sensitivity of animals to cold. **E**, **F** Laser heat tingling and tail immersion test were used to detect the sensitivity of rats to a thermal stimulus. The results were presented as means ± SD from 8 rats in each group, n = 8. ^##^p < 0.01, ^#^p < 0.05 compared with the blank group; *p < 0.05, **p < 0.01 compared with paclitaxel model group

In this experiment, paclitaxel caused obvious mechanical, cold and thermal allodynia and hyperalgesia in rats (Fig. 2a), implying peripheral neuropathy in rats. During this experiment period, there was no death occurred. The body weight of rats showed an increasing trend and did not exhibit significant differences among groups (Fig. 2b, p > 0.05).

Compared with the paclitaxel group, 30 mg/kg and 120 mg/kg CSBTA group gradually recovered mechanical threshold from the 20th and the 16th day (Fig. 2c, **p < 0.01). There was no significant difference in mechanical threshold between the 120 mg/kg CSBTA group and the blank group after the 24th day by Von-Frey test (Fig. 2c, p > 0.05). 30 mg/kg or 120 mg/kg CSBTA groups gradually improved cold pain threshold from the 20th and the 18th day (Fig. 2d, **p < 0.01), and there was no significant difference between 120 mg/kg CSBTA group and blank group after 20 days (Fig. 2d, p > 0.05).

The response to thermal sensitivity was measured by laser thermal pain meter and tail immersion assay. Compared with the thermal sensitivity in the paclitaxel group (Fig. 2e), 30 mg/kg or 120 mg/kg CSBTA recovered the thermal threshold from the 16th day (**p < 0.01) in the laser thermal pain experiment. In comparison, the 30 mg/kg and 120 mg/kg CSBTA group exhibited no distinct difference compared with the blank group (p > 0.05) from the 24th and the 22nd day. In the tail immersion experiment (Fig. 2f), 30 mg/kg and 120 mg/kg CSBTA restored thermal sensitivity from the 22nd and the 18th day (**p < 0.01). Compared with the blank group, the 120 mg/kg CSBTA group already had no significant difference after the 24th day (p > 0.05). On the 40th day, 120 mg/kg CSBTA group and 30 mg/kg CSBTA group had a significant difference in mechanical pain thresholds (P < 0.01), but there were no significant differences in thermal sensitivity thresholds (P > 0.05). All these results indicated that CSBTA made neurological behaviors of PIPN gradually recover to normal.

### CSBTA decreased the levels of IL-1β, TNF-α, PGE2, and neuropeptides in PIPN rats

**Fig. 3:**
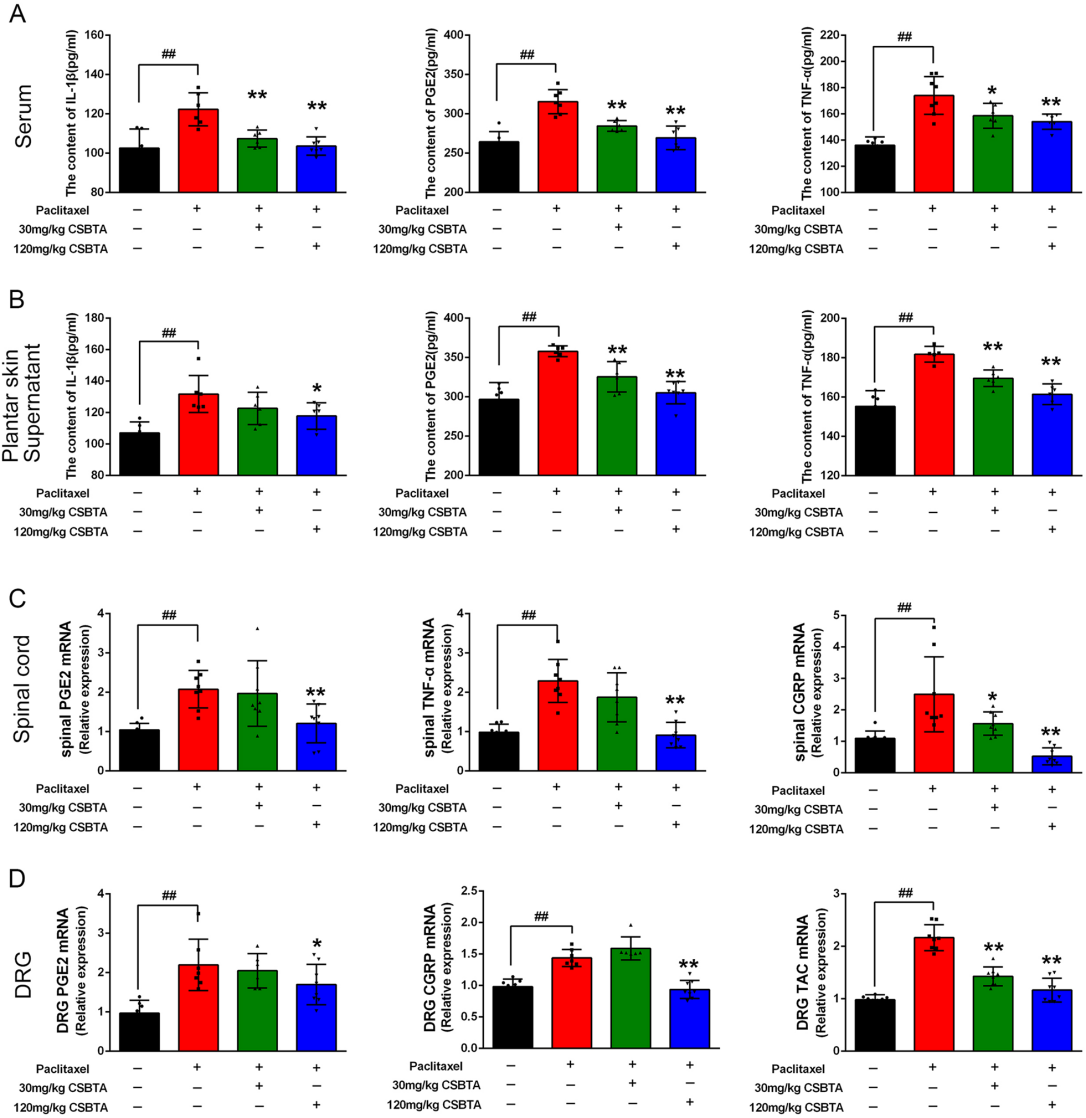
Effects of CSBTA on PIPN-induced pro-inflammatory cytokines release and gene expression in serum and tissues of rats. The release of IL-1β (left), PGE2 (middle), and TNF-α (right) in serum (**A**) and plantar skin supernatant (**B**) (n = 6). **C** The gene expression of pro-inflammatory cytokines in the spinal cord (**C**) and DRG (**D**) (n = 8). ^##^p < 0.01, ^#^p < 0.05 compared with the blank groups; *p < 0.05, **p < 0.01 compared with paclitaxel group

The inflammatory process is considered a potential trigger for chemotherapy-induced peripheral neuropathy. The release of pro-inflammatory cytokines or chemokines is regarded as the major mechanism of neuro-immune response [2, 37]. We measured the levels of inflammatory factors IL-1β, TNF-α, and PGE2 in serum and plantar skin supernatant. As shown in Fig. 3a and b, cytokines were significantly increased in the paclitaxel group compared with the blank group (##p < 0.01). However, CSBTA (30 mg/kg) reduced cytokines to some extent (*p < 0.05), while CSBTA (120 mg/kg) presented more obvious improvements (**p < 0.01), especially in the contents of serum IL-1β and plantar skin supernatant TNF-α.

SP and CGRP are excitatory neuropeptides released by the primary sensory afferent nerve in the dorsal horn [38]. Several studies have reported that SP and CGRP released from the spinal cord and DRG were involved in PIPN [39]. The results indicated that paclitaxel significantly increased the levels of inflammatory factors (PGE2, TNF-α) and neuropeptides (CGRP and SP, SP is synthesized by tachykinin, TAC-1) in the spinal cord and DRG compared with the blank group (Fig. 3c and d, #p < 0.05 or ## p < 0.01), while 120 mg/kg CSBTA group exerted an inhibitory effect on the mRNA levels of those factors (*p < 0.05 or **p < 0.01). Compared with 30 mg/kg CSBTA, 120 mg/kg CSBTA has a better inhibitory effect on cytokines, such as CGRP and TNF-α in the spinal cord, and PGE2 in DRG.

### CSBTA inhibited PKCε/p38 MAPK/TRPV1 pathway in the spinal cord and DRG

**Fig. 4:**
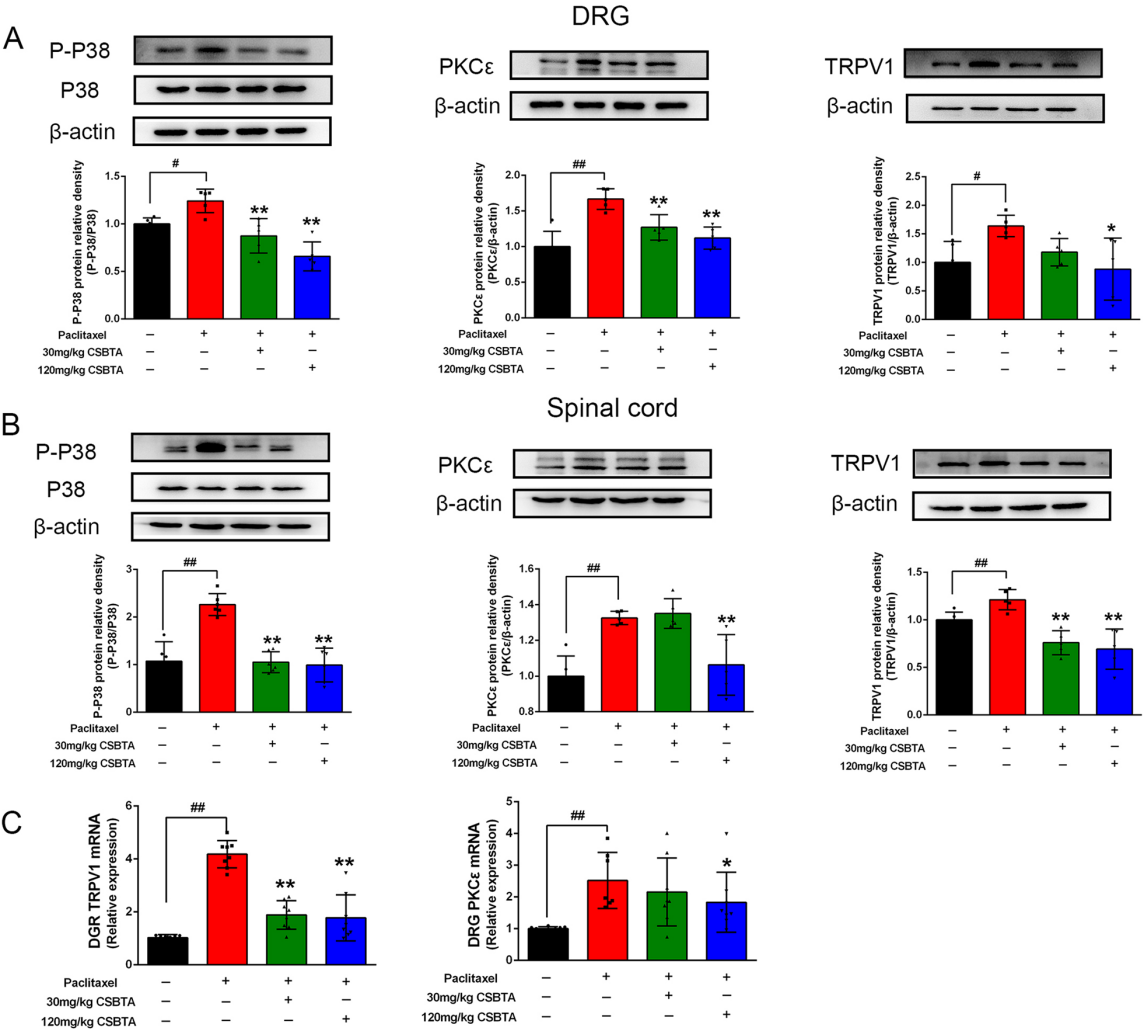
Effects of CSBTA on PIPN-induced protein and gene expression in different tissues of rats. Representative Western blots of p-p38 MAPK (left), PKCε (middle), TRPV1 (right) expression in PIPN rats (n = 5). The gene expression of TRPV1 and PKCε in DRG (**D**) of PIPN rats (n = 6). The corresponding quantitative data were present as mean ± SD. ^#^p < 0.05, ^##^p < 0.01 compared to the blank group, *p < 0.05, **p < 0.01 compared to the paclitaxel group

Western-blot and RT-qPCR analyzed the protein and mRNA expressions of PKCε, TRPV1, p-p38 MAPK from the spinal cord and DRG. CSBTA could significantly inhibit protein levels of PKCε, p-p38 MAPK, TRPV1 (Fig. 4, *p < 0.05 or **p < 0.01). Compared with 30 mg/kg, 120 mg/kg CSBTA had more significant inhibition on PKCε in the spinal cord (P < 0.05). There is no significant difference in the DRG between 30 mg/kg and 120 mg/kg CSBTA groups (P > 0.05). The mRNA levels of TRPV1 and PKCε were increased in DRG tissues of the paclitaxel group, whereas 120 mg/kg CSBTA significantly inhibited the expression of these genes.

According to the above results, the preliminary assumption was that CSBTA reduced the levels of pro-inflammatory cytokines (such as IL-1β, TNF-α, PGE2) and alleviated the symptoms of chronic neuropathic pain via regulating PKCε/p38 MAPK/TRPV1 pathway in PIPN rats. In order to confirm the mechanism of CSBTA on the amelioration of PIPN, we conducted the following experiments in vitro.

### CSBTA reduced the PGE2, TNF-α, and CGRP mRNA levels in paclitaxel-stimulated primary DRG neurons

**Fig. 5:**
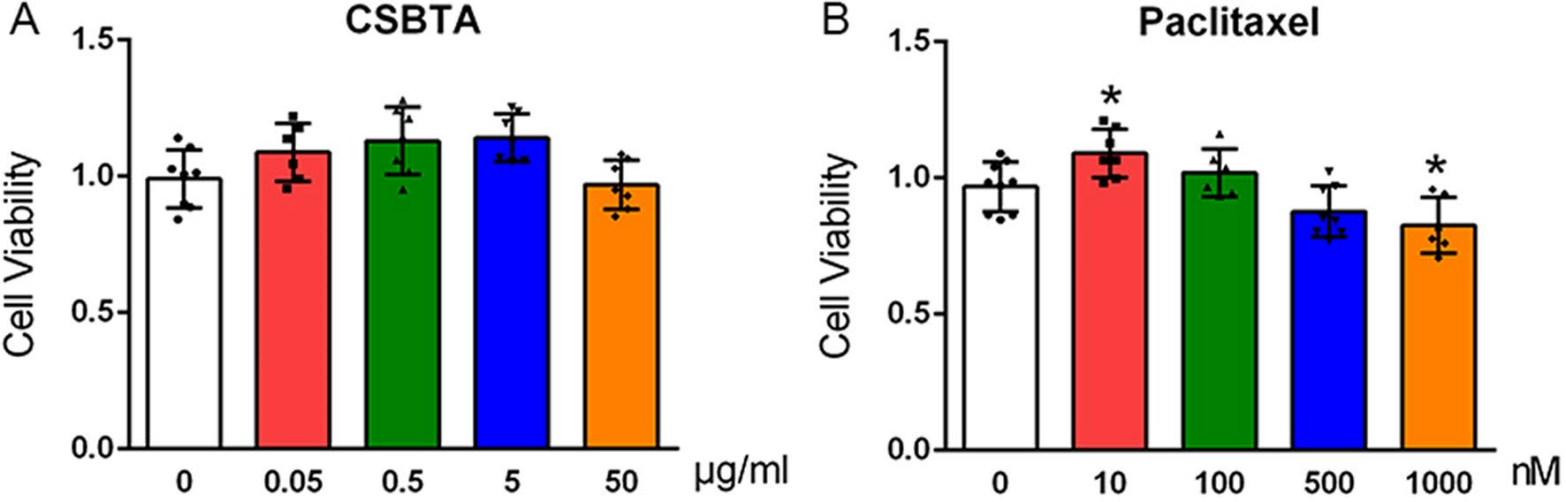
Effects of CSBTA and paclitaxel on primary DRG neurons viability. At 4th days, CSBTA (0, 0.05, 0.5, 5, 50 μg/ml) and paclitaxel (0, 10, 100, 500, 1000 nM) were administered for 5 days, respectively. The absorbance value at 450 nm was measured. The data were presented as means ± SD, n = 6. ^##^p < 0.01, ^#^p < 0.05 compared with the blank group; *p < 0.05, **p < 0.01 compared with paclitaxel group

Primary DRG neurons were incubated with a series of CSBTA and paclitaxel concentrations for 5 days, and the CCK-8 measured cell viability. As shown in Fig. 5, 300 nM paclitaxel and 50 μg/ml CSBTA did not significantly affect cell viability (p > 0.05), which are suitable concentration in vitro experiments.

**Fig. 6:**
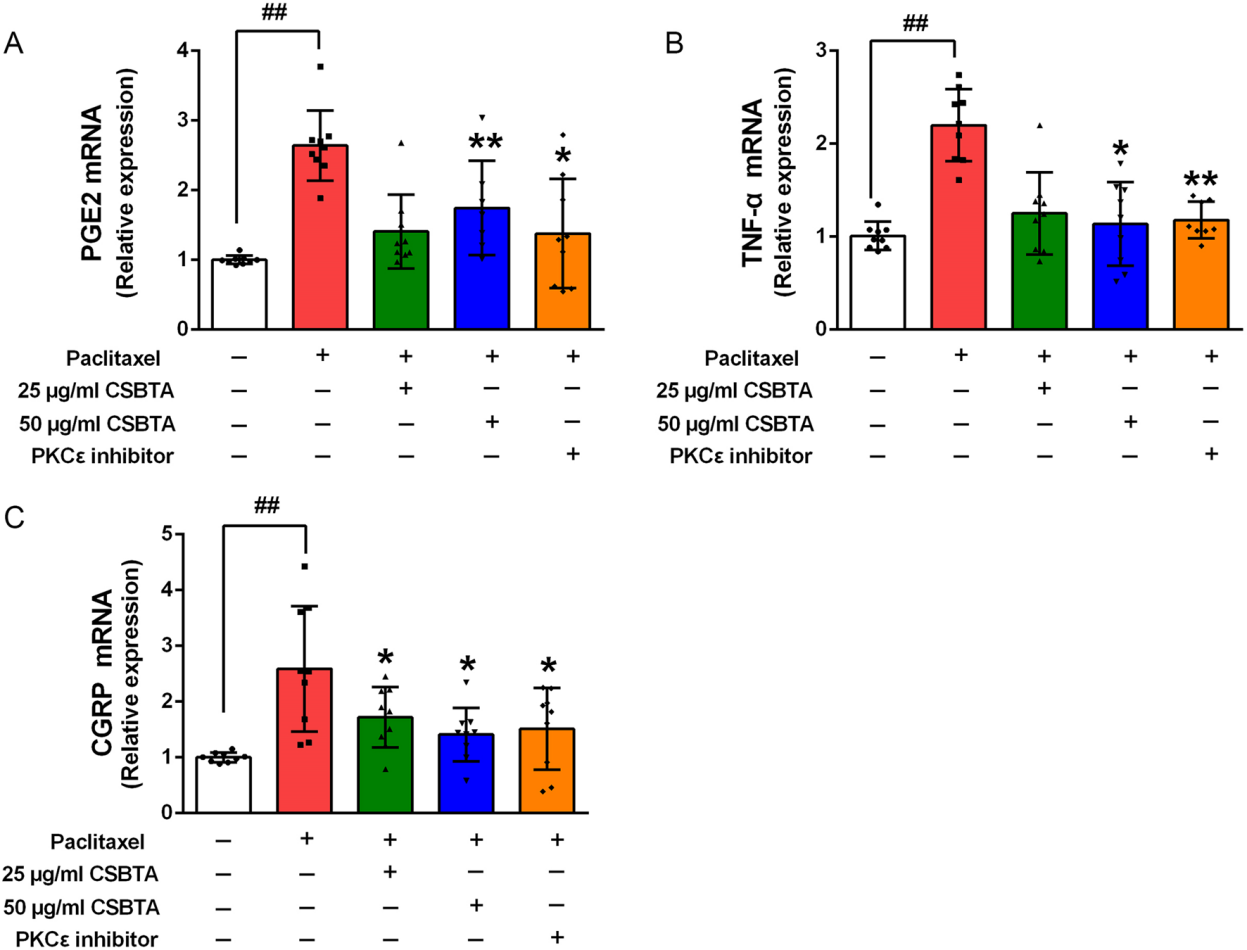
Effects of CSBTA on PGE2 (**A**), TNF-α (**B**), and CGRP (**C**) mRNA levels in paclitaxel-stimulated primary DRG neurons. The data were presented as means ± SD, n = 9. ^##^p < 0.01, ^#^p < 0.05 compared with the blank group; *p < 0.05, **p < 0.01 compared with paclitaxel group

Paclitaxel up-regulated the expression of PGE2, TNF-α, and CGRP in primary DRG neurons (Fig. 6, ##p < 0.01). These cytokines could be inhibited by 25 μg/ml CSBTA, 50 μg/ml CSBTA, and 100 nM staurosporine. 50 μg/ml CSBTA significantly repressed the gene expressions of PGE2, TNF-α, and CGRP compared with those in the paclitaxel group (*p < 0.05 or **p < 0.01).

### CSBTA regulated the PKCε/p38 MAPK/TRPV1 pathway in paclitaxel-stimulated primary DRG neurons

**Fig. 7:**
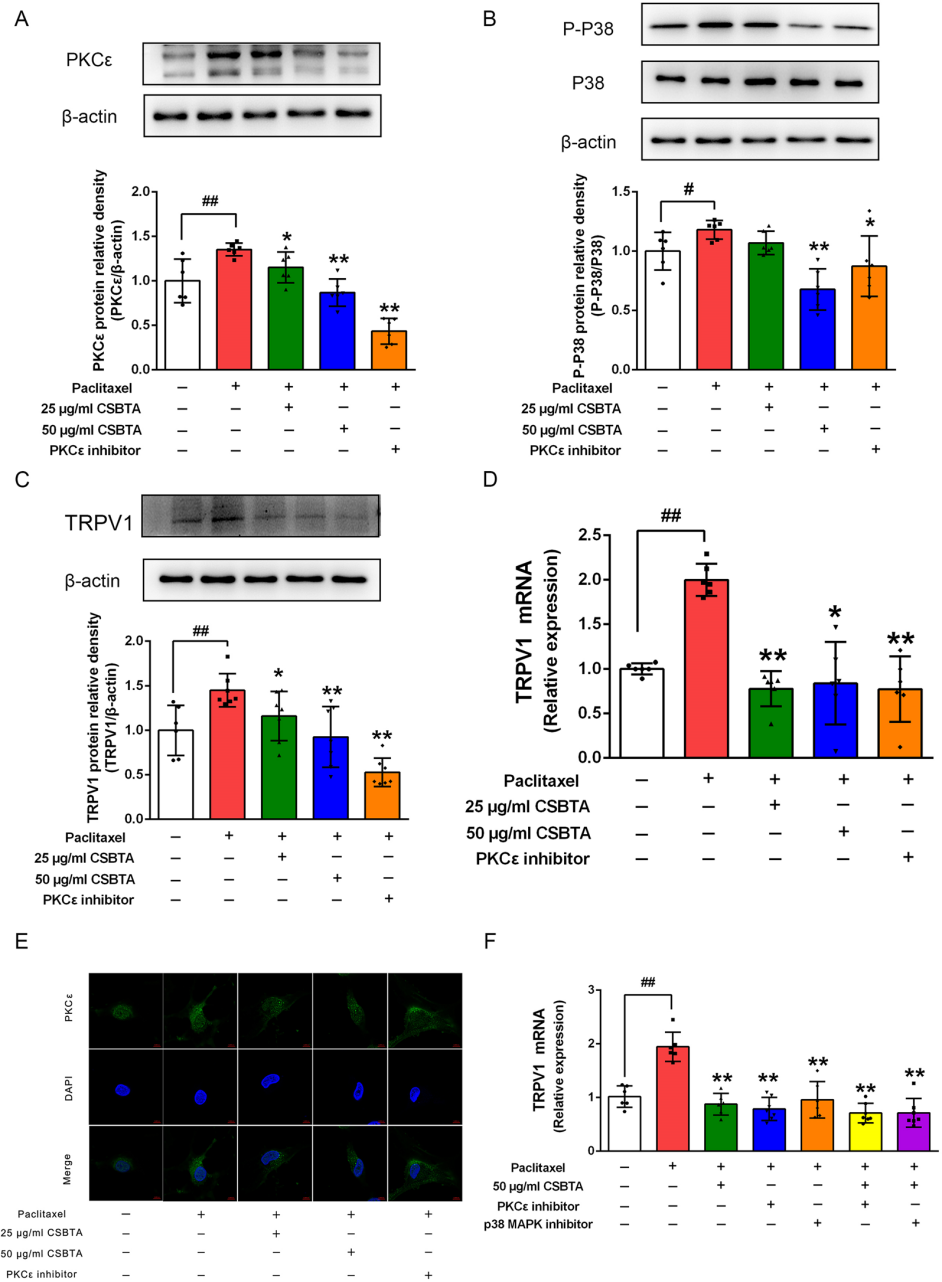
Effects of CSBTA on the protein expression of PKCε (**A**), p-p38 MAPK (**B**), and TRPV1 (**C**) and the TRPV1 mRNA levels (**D**, **F**) in paclitaxel-stimulated primary DRG neurons. The corresponding quantitative data were present as mean ± SD. n = 6. **E** Representative images of PKCε (green) and DAPI (blue) stained DRG neuron. Scale bar, 5 μm. ^#^p < 0.05, ^##^p < 0.01 compared with the blank group, *p < 0.05, **p < 0.01 compared with the paclitaxel group

In Fig. 7, the paclitaxel group significantly increased the expressions of PKCε (a), p-p38 MAPK (b) and TRPV1 protein (c) and TRPV1 mRNA level (d) in primary DRG neurons compared with the blank group (##p < 0.01), whereas CSBTA (50 μg/ml) and staurosporine (100 nM) could significantly reduce relevant protein expressions and TRPV1 mRNA level (**p < 0.01). In line with the idea that CSBTA treatment decreases the PKCε expression, paclitaxel increased the fluorescence intensity of PKCε in primary DRG neurons, and this effect was suppressed by 50 μg/ml CSBTA and 100 nM staurosporine (Fig. 7e).

As shown in Fig. 7a–e, staurosporine, as a PKCε inhibitor, not only significantly blocked the increase of PKCε protein expression induced by paclitaxel but also obviously inhibited the paclitaxel-induced p38 MAPK phosphorylation as well as the protein and mRNA levels of TRPV1. In order to further explore the relationship between PKCε, p38 MAPK, and TRPV1, we used the p38 MAPK inhibitor (SB203580) to observe whether blocking p38 MAPK could reduce the TRPV1 mRNA level in the PIPN model. Figure 7f showed that blocking PKCε or p38 MAPK could significantly inhibit the mRNA level of TRPV1. In addition, CSBTA was combined with PKCε inhibitor or p38 MAPK inhibitor to confirm the role of CSBTA. However, there were no significant differences in TRPV1 mRNA level between the combination of 50 μg/ml CSBTA and PKCε or p38 MAPK inhibitors and the use of each inhibitor alone. These results suggested that CSBTA probably achieved an analgesic effect by inhibiting PKCε/p38 MAPK/TRPV1 signaling pathway.

## Discussion

Paclitaxel-induced peripheral neuropathy in rats is regarded as an internationally recognized animal model of neuropathic pain. PIPN model produces persistent pain behaviors, including mechanical hyperalgesia and temperature hyperalgesia (heat and cold), which will contribute to elucidate the mechanisms [6, 40]. In the present study, through intraperitoneal injections of paclitaxel (2 mg/kg), the mechanical and thermal allodynia and hyperalgesia in rats were changed. As shown in Fig. 2, 30 mg/kg CSBTA and 120 mg/kg CSBTA gradually recovered mechanical pain caused by paclitaxel after 2 weeks and eventually normalized. Some studies have shown that paclitaxel could induce thermal hyperalgesia in the PIPN rat model, consistent with clinical hyperalgesia in patients with peripheral neuropathy [6, 41]. We used a thermal laser instrument and a tail-flick test to detect the heat sensitivity of rats. The results showed that 30 mg/kg CSBTA and 120 mg/kg CSBTA could normalize thermal hyperalgesia of the foot and tail. Cold-induced pain is often used as an indicator to measure neuropathic pain behavior in animal models. Detection of cold-induced pain is an important method to study the underlying pathophysiological mechanism of pain. In rodents, there are various methods for detecting cold-induced pain, such as acetone, cold plate experiments, and ethyl chloride spray. Each test has its advantages and disadvantages [42]. In this experiment, we used dry ice to stimulate the feet of rats. The investigation found that 30 mg/kg CSBTA and 120 mg/kg CSBTA restored the threshold of cold hyperalgesia in PIPN rats.

TRPV1, one of the nonselective cation channels, could be activated by heat (43 ℃), protons, and ligand (e.g., capsaicin) [6, 43]. TRPV1 is transported from DRG along the sciatic nerve to peripheral nerves of the skin and is also transferred from DRG to the dorsal horn of the spinal cord [16]. Hara et al*.* [7] have confirmed that intraperitoneal injection of paclitaxel could increase TRPV1 in rat plantar skin and sensitize the TRPV1 channel, which is regarded as the cause of heat hyperalgesia. Some studies have shown that intraperitoneal injection of TRPV1 antagonists (such as capsazepine and SB366791) in PIPN animals would attenuate paclitaxel-induced thermal hyperalgesia in a dose-dependent manner but exhibited no pronounced effect on mechanical allodynia [6], further confirming the relationship between TRPV1 and thermal hyperalgesia. Inflammatory states or injection of some cytokines (such as bradykinin and nerve growth factor) could develop into severe thermal hyperalgesia, and TRPV1^−/−^ mice would provide genetic support for the idea that TRPV1 is a crucial component of inflammation-induced hyperalgesia [44]. In combination with the current animal experiments and relevant clinical studies of pain, we found no particularly appropriate, available, and universally recognized TRPV1 antagonist in the clinical treatment of pain. Therefore, we did not set a positive drug group in the animal part of this experiment. Activation of TRPV1 led to depolarization of neurons and the release of neuropeptides in peripheral and central nerve endings. And then, neuropeptides are coupled with their related receptors and enhance the sensitivity of nociceptors [12]. Excitatory neuropeptides, such as calcitonin gene-related peptides (CGRP), are released from primary afferent nociceptors, which are crucial in initiating and developing pain. It can be recognized as a potential biomarker for the activation of sensory neurons [45]. The increased release of neuropeptides induced by paclitaxel may result in sensitization of primary sensory neurons and subsequently caused mechanical allodynia and tingling in patients [46]. Therefore, we used CGRP mRNA as an indicator to observe the paclitaxel effect on neuronal function.

The increase of PKC expression in DRG was observed in the PIPN mouse model, and inhibition of PKC attenuated paclitaxel-induced chronic neuropathic pain [13]. There are three isoforms of PKC that are co-labeled with TRPV1 on nociceptors [6, 45]. Among these isoforms, PKCε is the most important in paclitaxel-induced neuropathy [47]. PKCε, one of the subtypes of PKC, induces intracellular signaling pathways in primary nociceptor receptors and mediates cytokine-induced nociceptor receptors activation. It is responsible for the transition from acute to chronic pain [45]. PKCε is also a kind of the second messenger, which mediates paclitaxel-induced hyperalgesia [21]. Recent studies have shown that one of the targets of PKCε in peripheral nociceptive signaling is TRPV1 [45, 48–50]. A variety of inflammatory mediators (e.g., ATP, bradykinin, and prostaglandins) could enhance TRPV1 through the PKC-dependent pathway and reduce the temperature threshold of TRPV1 [21]. Activation of PKC, especially PKCε, sensitizes nociceptor responses, but PKC inhibitors or mutations can attenuate these responses by disrupting the phosphorylation sites of TRPV1 [51]. Zhang and Mcnaughton proposed that inhibition of plasma-membrane translocation of PKCε could also ameliorate paclitaxel-induced peripheral neuropathy [52, 53]. In addition, TRPV1 activation regulates the excitatory synaptic transmission of sensory synapses, and the activation of PKC can further enhance its effect. This finding provides a potential mechanism that sustained activation of PKC will contribute to hyperalgesia in PIPN [54].

Mitogen-activated protein kinases (MAPK), mediated by p38 rather than ERK, acts on DRG neurons in the pathogenesis of pain [16]. Previous studies have illustrated that p38 MAPK plays an important role in mechanical allodynia and thermal hyperalgesia after nerve injury, and expression of p-p38 MAPK was elevated in DRG neurons after chronic constriction injury of the sciatic nerve [55–57]. In general, p38 MAPK is a crucial regulator in DRG neurons and spinal cord neurons, involving in the progression of PIPN. TRPV1 is a heat-sensitive receptor in DRG neurons, and its expression is upregulated after sensory nerve damage, related to activation of p38 MAPK in primary sensory neurons [16, 58]. p38 MAPK activation in DRG would enhance the expression of TRPV1 and promote TRPV1 translation and transporting to peripheral sensors, which is helpful to maintain heat hyperalgesia [43]. In addition, the p38 MAPK pathway directly or indirectly activates transcription factors and induces the release of pro-inflammatory cytokines such as TNF-α, IL-1β, and IL-6 [16]. Studies have found that increased expression of p-p38 MAPK and TRPV1 in the peripheral and central nervous systems is usually accompanied by increased pro-inflammatory cytokines in the spinal dorsal horn [59]. Ji et al. found that activation of p38 MAPK in the DRG increases TRPV1 levels in nociceptor peripheral terminals and contributes to the maintenance of inflammatory pain [16]. Whereas TRPV1 coexists with p38 MAPK in microglia and excitatory neurons [59], the axonal transport of TRPV1 may be related to the phosphorylation and activation of p38 MAPK in the inflammatory environment. Inhibition of p-p38 MAPK expression can significantly reduce the content of TRPV1 in the neuronal body and skin tissue and alleviate the temperature sensitivity caused by inflammation [16, 60]. Therefore, thermal nociceptive sensitivity can be regulated via p38 MAPK/TRPV1 in the peripheral and central nervous systems in physiological and pathological conditions [59, 61].

Paclitaxel does not penetrate the blood–brain barrier and could not accumulate in the central nervous system but will damage the peripheral nervous system, including nerve endings, peripheral nerves, and DRG. Paclitaxel causes damage to the sciatic nerve, nerve endings, and DRG during the development of PIPN [13, 62]. The results showed that 120 mg/kg CSBTA could effectively inhibit PKCε, p-p38 MAPK, and TRPV1 protein expressions (Fig. 4). Accordingly, paclitaxel increased the level of inflammatory cytokines (such as TNF-α, IL-1β, and PGE2 in serum and plantar skin supernatant) in PIPN, and CSBTA exhibited significant inhibition on pro-inflammatory cytokines. In the spinal cord and DRG tissues, gene levels of PGE2, TNF-α, CGRP, and SP showed the same situation (Fig. 3). These results are consistent with the published results of CSBTA improving cisplatin-induced peripheral neuropathy [25]. According to the in vivo experiments, we found that CSBTA alleviates the inflammatory state by inhibiting PKCε, p-p38 MAPK, and TRPV1 protein levels and affects related behavioral indicators to achieve the purpose of ameliorating PIPN eventually.

DRG is isolated from the blood–brain barrier and is surrounded by rich capillaries, making it highly susceptible to drug neurotoxicity and participating in sensory signal transmission [63]. Although paclitaxel doesn’t penetrate the blood–brain barrier, it can accumulate in dorsal root ganglion neurons [64]. The concentration of paclitaxel and CSBTA did not affect cell viability (Fig. 5). Consistent with the data obtained from animal experiments, 50 μg/ml CSBTA significantly inhibited protein contents of PKCε, p-p38 MAPK, and TRPV1, and reduced mRNA levels of PGE2, TNF-α, and CGRP (Figs. 6 and 7). Interestingly, inhibitors of PKCε reduced cytokines production, suggesting that paclitaxel increased cytokines by activating PKCε. Although the inhibitory effect of PKCε inhibitors on TRPV1 and PKCε protein is stronger than the inhibitory effect of CSBTA, 50 μg/ml CSBTA has a more obvious downregulation of p-p38 MAPK, suggesting that CSBTA has multiple targets.

**Fig. 8:**
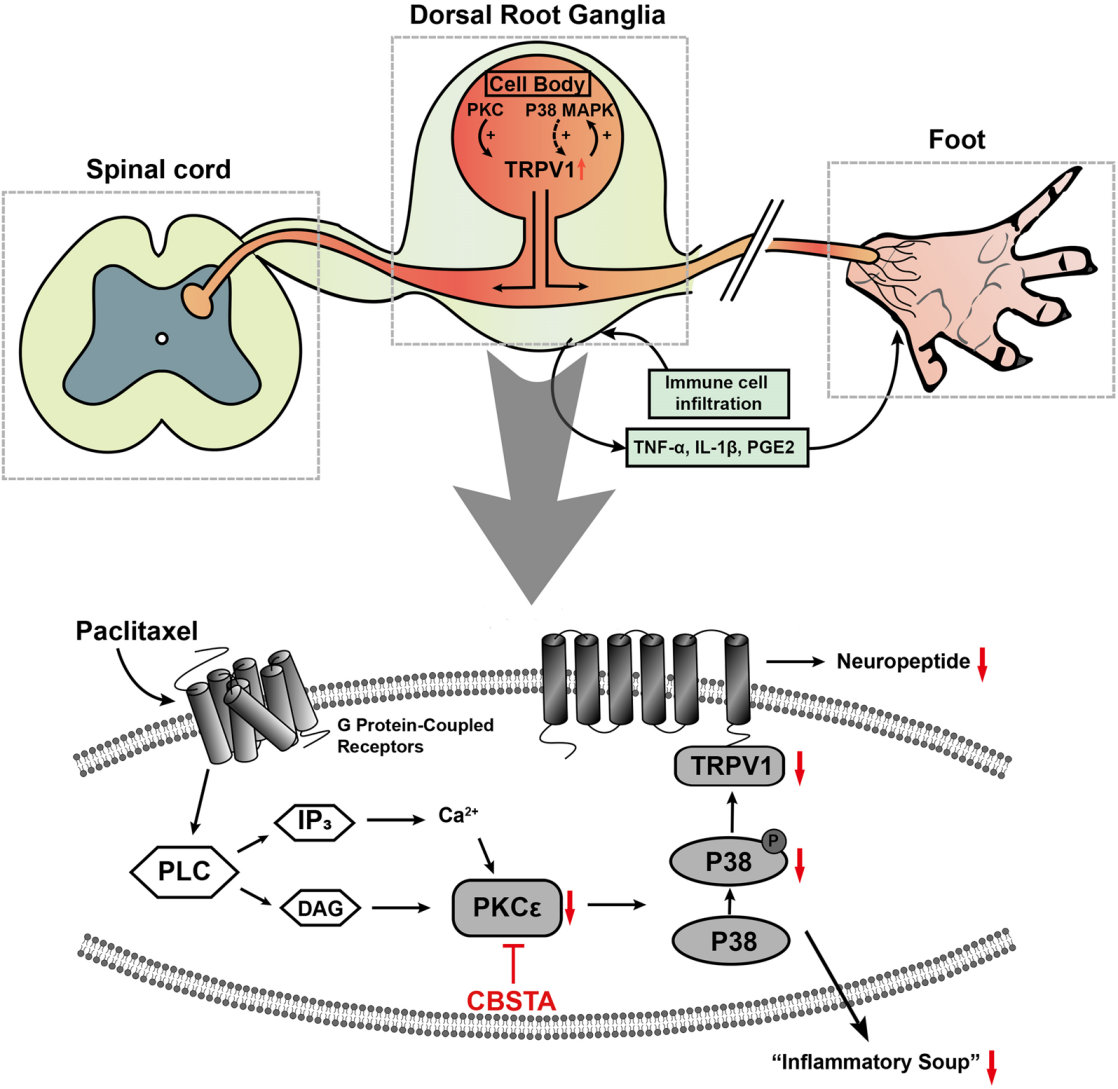
Schematic summary of CSBTA effect and potential mechanism on PIPN

Previous studies showed that Ca^2+^-dependent PKC-MAPK pathways, including the ERK, JNK, and p38-type signaling pathways, participate in cardiac remodeling, visceral leishmaniasis, oxidative stress, immunomodulatory effect, and etc. [65–68] As a downstream molecule of Ca^2+^-dependent signaling pathways, PKC is a ubiquitous serotonin protein kinase that catalyzes the phosphorylation of many cell proteins [67]. Additionally, the activation of MAPK is recognized as a pathogenic mechanism in chronic pain [69]. In recent years, exploring the multiple functions of signaling pathways and interactions between targets have gradually become a research focus, and PKC/p38 MAPK is no exception. Overall, those studies indicate that PKC/p38 MAPK pathway has multiple functions [70, 71]. At the same time, numerous studies demonstrated that PKC is an upstream molecule that regulates the activation of p38 MAPK in the PIPN process [15, 65]. In combination with the PKCε inhibitor in the PIPN cell model, it could significantly inhibit the protein content of p38 MAPK (Fig. 7b) and reduce the gene expression of inflammatory factors (Fig. 6). Thus, PKCε was an upstream molecule of p38 and TRPV1 in PIPN cell models to improve the inflammation state of DRG neurons. Next, we examined whether the p38 MAPK pathway regulates TRPV1 and found that the p38 inhibitor SB203580 significantly suppressed paclitaxel-induced upregulation of TRPV1 in vitro (Fig. 7f). Previous research mentioned that p38 activation in small to medium-sized DRG neurons might have a crucial role in the expression of TRPV1 [16, 72]. Thus, our finding that paclitaxel-induced p38 MAPK has an essential role in the TRPV1 channel is not surprising. In addition, there was no significant difference in TRPV1 expression between CSBTA combined with SB203580 and SB203580 alone. In addition, there was no significant difference in TRPV1 mRNA level between the combination of CSBTA and staurosporine and staurosporine alone. Our data suggested that CSBTA probably achieved an analgesic effect by inhibiting PKCε/p38 MAPK/TRPV1 signaling pathway (Fig. 8).

## Conclusions

Overall, CSBTA effectively improved behavioral indexes of mechanical and thermal allodynia and hyperalgesia and regulated the contents of pro-inflammatory cytokines (such as IL-1β, TNF-α, PGE2) and neuropeptides (SP and CGRP) in different tissues in vivo. We concluded that CSBTA could ameliorate pain behavior and the inflammatory status of PIPN. CSBTA decreased relevant protein expressions in PKCε/p38 MAPK/TRPV1 signaling pathway in the spinal cord and DRG of PIPN rats and primary DRG neurons (Fig. 8). Therefore, CSBTA has a perspective therapeutic effect on the treatment of paclitaxel-induced peripheral neuropathy.

## Data Availability

Not applicable.

## Abbreviations

CGRP: Calcitonin gene-related peptide
CIPN: Chemotherapy-induced peripheral neuropathy
CSBTA: Corydalis saxicola Bunting total alkaloids
DRG: Dorsal root ganglion
IL-1β: Interleukin-1β
PGE2: Prostaglandin E2
PIPN: Paclitaxel-induced peripheral neuropathy
PKCε: Protein kinase C ε
SP: Substance P
TNF-α: Tumor Necrosis Factor-α
TRPV1: Transient receptor potential vanilloid 1
